# Microglial TREM2 and cognitive impairment: insights from Alzheimer’s disease with implications for spinal cord injury and AI-assisted therapeutics

**DOI:** 10.3389/fncel.2025.1705069

**Published:** 2025-11-07

**Authors:** Zhonghan Wu, Shuisheng Yu, Dasheng Tian, Li Cheng, Juehua Jing

**Affiliations:** 1 Department of Orthopaedics, The Second Affiliated Hospital of Anhui Medical University, Hefei, China; 2 Institute of Orthopaedics, Research Center for Translational Medicine, The Second Affiliated Hospital of Anhui Medical University, Hefei, China

**Keywords:** Alzheimer’s disease, artificial intelligence, cognitive impairment, microglia, spinal cord injury, TREM2

## Abstract

Cognitive impairment is a frequent but underrecognized complication of neurodegenerative and traumatic central nervous system disorders. Although research on Alzheimer’s disease (AD) revealed that microglial triggering receptor expressed on myeloid cells 2 (TREM2) plays a critical role in inhibiting neuroinflammation and improving cognition, its contribution to cognitive impairment following spinal cord injury (SCI) is unclear. Evidence from AD shows that TREM2 drives microglial activation, promotes pathological protein clearance, and disease-associated microglia (DAM) formation. SCI patients also experience declines in attention, memory, and other functions, yet the specific mechanism of these processes remains unclear. In SCI, microglia and TREM2 are involved in inflammation and repair, but their relationship with higher cognitive functions has not been systematically examined. We infer that TREM2 might connect injury-induced neuroinflammation in the SCI with cognitive deficits, providing a new treatment target. Artificial intelligence (AI) offers an opportunity to accelerate this endeavor by incorporating single-cell transcriptomics, neuroimaging, and clinical data for the identification of TREM2-related disorders, prediction of cognitive trajectories, and applications to precision medicine. Novel approaches or modalities of AI-driven drug discovery and personalized rehabilitation (e.g., VR, brain–computer interface) can more precisely steer these interventions. The interface between lessons learned from AD and SCI for generating new hypotheses and opportunities for translation.

## 1 Introduction

Cognitive impairment is a common complication in neurodegenerative and traumatic CNS (CNS) disorders (Jessen et al., 2014; Wang C. et al., 2022; Mathys et al., 2023). Memory, attention, and executive impairments in Alzheimer’s disease (AD) are well-known phenomena strongly contributing to the overall disease process (Leuzy et al., 2024; Nasb et al., 2024; Peña-Bautista et al., 2024; Reyes et al., 2025). Also, spinal cord injury (SCI) patients often suffer from problems with attention, learning, and memory, which affect the ability to rehabilitate and life quality (Pasipanodya et al., 2021; Shabany et al., 2022; Vaccaro et al., 2022; Yang et al., 2023; Li Y. et al., 2024; Welkamp et al., 2024). These findings indicate cognition to have a critical impact on neurological outcome in various situations.

As the resident immune cells of the CNS, microglia are important in shaping cognitive functions (Haure-Mirande et al., 2022; Shi K. et al., 2022; Shi Q. et al., 2022). Besides immune surveillance, they are involved in synaptic pruning, neurogenesis, and circuit remodeling (Bellver-Landete et al., 2019; Zhou et al., 2020; Brennan et al., 2022; Choi et al., 2023). Impaired microglial activity has been proposed to be linked to impairment of cognitive function, indicating that immune–neural interaction may be a shared mechanism common in different diseases (Zhang et al., 2022).

One major advance is the identification of TREM2 (triggering receptor expressed on myeloid cells 2) as a key regulator of microglial functions (Haure-Mirande et al., 2022; Shi K. et al., 2022; Shi Q. et al., 2022). In AD, genetic variants in TREM2 increase disease risk, and functional studies show that TREM2 signaling promotes microglial responses, enhances clearance of pathological proteins, and influences cognitive outcomes (Jiang et al., 2014; Fracassi et al., 2023; Huang et al., 2023; Li et al., 2023). These findings highlight TREM2 as both a mechanistic driver and a therapeutic target.

Cognitive impairment in SCI gradually receives more attention, although the mechanisms of the process are not well understood (Craig et al., 2017; Sachdeva et al., 2018; Li et al., 2020; Alcántar-Garibay et al., 2022). Research has mostly focused on systemic inflammation, chronic pain, and mood disorders (Jure and Labombarda, 2017; Molina-Gallego et al., 2024), and the role of unique immune pathway proteins such as TREM2 has been less well researched (Craig et al., 2017; Sachdeva et al., 2018; Li et al., 2020; Alcántar-Garibay et al., 2022). This gap restricts our mechanistic knowledge and therapy development.

The question at the core of this review is whether mechanistic insights from AD, including those addressing microglial TREM2, may inform pathways leading to cognitive dysfunction post-SCI. Cognitive impairment following SCI has been stably demonstrated in both animal and human studies (Craig et al., 2017; Sachdeva et al., 2018; Li et al., 2020; Alcántar-Garibay et al., 2022), with activation of microglia in the hippocampus and prefrontal cortex implicated in the induction of a chronic, low-grade neuroinflammatory state that impairs synaptic homeostasis and neuronal plasticity (Jure et al., 2017; Yu et al., 2024). TREM2 mutations clearly lead to cognitive decline in AD patients (Jiang et al., 2014; Fracassi et al., 2023; Li et al., 2023). Yet again, sensitizing evidence directly connecting TREM2 mutations with post-SCI cognitive impairments is still absent; such linkage currently constitutes an inferred hypothesis based on the mechanistic overlap of AD and SCI. In line with this, the present review will (1) bring together consolidated knowledge from AD and SCI concerning microglial TREM2 and cognition, and (2) comment on the role of artificial intelligence (AI) in assisting hypothesis generation and translational breakthroughs in this nascent field.

Here, we propose that the empirical knowledge in AD can provide important clues for exploring the role of the TREM2 signaling pathway-mediated microglial cell response in cognitive impairment following SCI. We also review the potential role of AI to expedite progress by integrating multimodal datasets, discovering therapeutic targets, and guiding individualized rehabilitation. Collectively, this framework can integrate neuroimmunology with cognition and computation for the development of translational strategies.

## 2 Cognitive dysfunction and microglial TREM2 in Alzheimer’s disease

### 2.1 Cognitive dysfunction in AD

To understand the potential contribution of TREM2 in SCI-related cognitive deficits, it is necessary to first summarize its established role in AD. Cognitive impairment is a typical symptom of AD, consisting most prominently of amnesia and executive deficits (Collij et al., 2024). Patients with AD commonly experience declines in encoding and retrieving information, problem-solving, as well as monitoring goal-directed behavior (Bäckman et al., 2004; Moguilner et al., 2024). These impairments are caused by neuropathological features such as tau pathology, synaptic loss, and network dysfunction, which mainly affect the integrity of cognitive circuits (Lin et al., 2022, 2025; Hu et al., 2024).

### 2.2 TREM2 as a regulator of microglial function in AD

TREM2 is a transmembrane receptor that is mainly expressed on microglia of the CNS, where it has an essential role in the regulation of microglial activation, phagocytosis, as well as inflammatory responses (Li et al., 2022; Li Z. et al., 2024; Yan et al., 2022). Through these functions, TREM2 promotes the ability of microglia to respond to neuronal injury and maintain CNS homeostasis (Kobayashi et al., 2016; Nugent et al., 2020).

**Figure 1:**
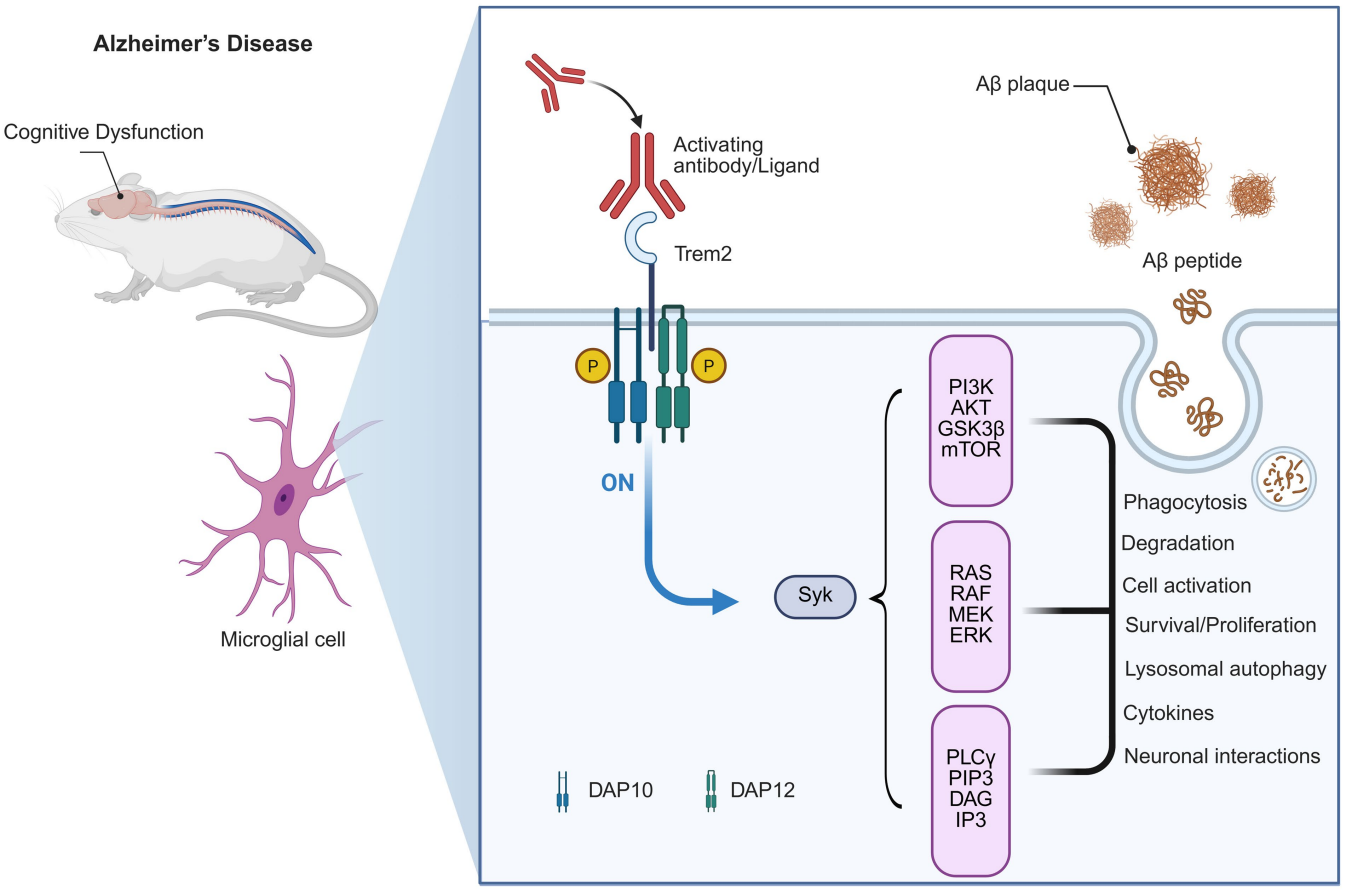
TREM2 signaling pathway in Alzheimer’s Disease and its link to cognitive dysfunction. This diagram illustrates the role of TREM2 in Alzheimer’s disease (AD) pathology and its association with cognitive dysfunction. In AD, activating antibodies or ligands (including amyloid-beta plaques) can activate TREM2. This activation triggers downstream signaling pathways, including the PI3K-AKT, RAS-RAF-MEK-ERK, and PLCγ pathways. These pathways are involved in critical cellular processes, such as Aβ plaque phagocytosis, degradation, and the modulation of neuroinflammation, all of which influence the progression of cognitive decline in AD. The activation of TREM2 and its signaling also plays a role in microglial cell activation and neuronal interactions, ultimately impacting the neurodegenerative process.

In AD, TREM2 has been identified as a critical regulator in neuroimmune processes that maintain cognitive function (Li et al., 2022). Functionally (Figure 1), TREM2 promotes the clearance of deposited amyloid-*β* (Aβ) plaques and relieves neuronal toxicity (Nugent et al., 2020). Additionally, TREM2 can drive the transformation of baseline microglia into “disease-associated microglia” (DAM) phenotype, which upregulates the phagocytosis of Aβ and other microglial responses (Nugent et al., 2020). These microglial responses are associated with maintenance of synaptic integrity and cognition in experimental models (Wang S. et al., 2022).

Genetic investigations further emphasize the importance of TREM2 for cognitive functions (Rachmian et al., 2024). Rare TREM2 variants are strongly associated with increased risk for AD, and individuals who carry these mutations show an earlier age of onset or more severe cognitive decline than noncarriers (Peng et al., 2023). Taken all together, these observations place TREM2 as both a key regulator of microglial activity and as a potential drug target to prevent cognitive impairment in AD (Jia et al., 2025).

## 3 Cognitive dysfunction and microglial TREM2 in spinal cord injury

### 3.1 Overlooked cognitive dysfunction after SCI

Based on AD findings, we next consider whether the analogous mechanisms may apply to SCI, while noting the limited direct evidence. Although SCI has conventionally been regarded as a disorder characterized by motor and sensory dysfunction, there is now accumulating literature to indicate that cognitive impairment is an important but under-recognized consequence (Craig et al., 2017; Sachdeva et al., 2018; Li et al., 2020; Alcántar-Garibay et al., 2022). Attention, working memory, and processing speed are often impaired in patients with SCI, which can interfere with rehabilitation and daily life quality (Craig et al., 2017; Sachdeva et al., 2018; Li et al., 2020; Alcántar-Garibay et al., 2022). In addition to the cognitive decline, such components as chronic pain, sleep disturbances, and mood disorders often worsen cognitive load and function outcome (Widerström-Noga, 2017; Eller et al., 2022; Wu et al., 2023).

The specific mechanisms of cognitive impairment after SCI remain poorly understood (Welkamp et al., 2024). Previous research in SCI has largely focused on systemic inflammation, secondary injury cascades, and psychosocial factors, and ignored molecular drivers of cognitive impairment (Brennan et al., 2022, 2024). Nevertheless, clinical neuroimaging evidence indicates that SCI may produce structural and functional changes in the brain, such as disrupted connections within prefrontal and hippocampal networks, which are important for attention, memory, and executive function (Jure and Labombarda, 2017; Welkamp et al., 2024). These data suggest that SCI has consequences beyond the damage in the spinal cord, affecting the wider networks that participate in cognitive processes.

Considering the dependence of microglia for modulating cognitive function in other neurologic diseases, it is possible that microglial pathways also participate in the impaired cognition process after SCI (Jure et al., 2017). In particular, molecules such as TREM2, which regulate microglial activation and synaptic remodeling in AD, may play a similar role in SCI (Gao et al., 2023; Zhao C. et al., 2025). Investigating TREM2-mediated pathological processes in the context of SCI could provide effective strategies for exploring the pathophysiology of cognitive impairment and identifying therapeutic targets as well as developing corresponding drugs for mitigating these often-overlooked deficits.

Clinical and neuroimaging studies suggest that SCI can bring substantial changes to brain network connectivity and functional activation patterns, specifically within regions implicated in attention and memory (Jure et al., 2022; Qin et al., 2025). Regardless of the causes, the mechanisms of cognitive impairment in AD and SCI may partially overlap (Jure et al., 2017; Oveisgharan et al., 2018). Substantial neuroinflammation can disrupt neural circuit remodeling and synaptic plasticity due to the prolonged activation of brain microglia (Jure et al., 2017; Oveisgharan et al., 2018; Doorduijn et al., 2019; Deng et al., 2024). Changes in neurotransmitter signaling, dendritic spine density, and synaptic connectivity also continue to hinder the ease of information transfer (Bäckman et al., 2004; Moguilner et al., 2024). Altogether, these findings suggest that immune-mediated synaptopathies and circuit changes may represent a common pathological substrate for impaired cognition in both neurodegenerative and traumatic CNS disorders.

### 3.2 TREM2 as a potential regulator in SCI-induced neuroinflammation and dysfunction

**Figure 2:**
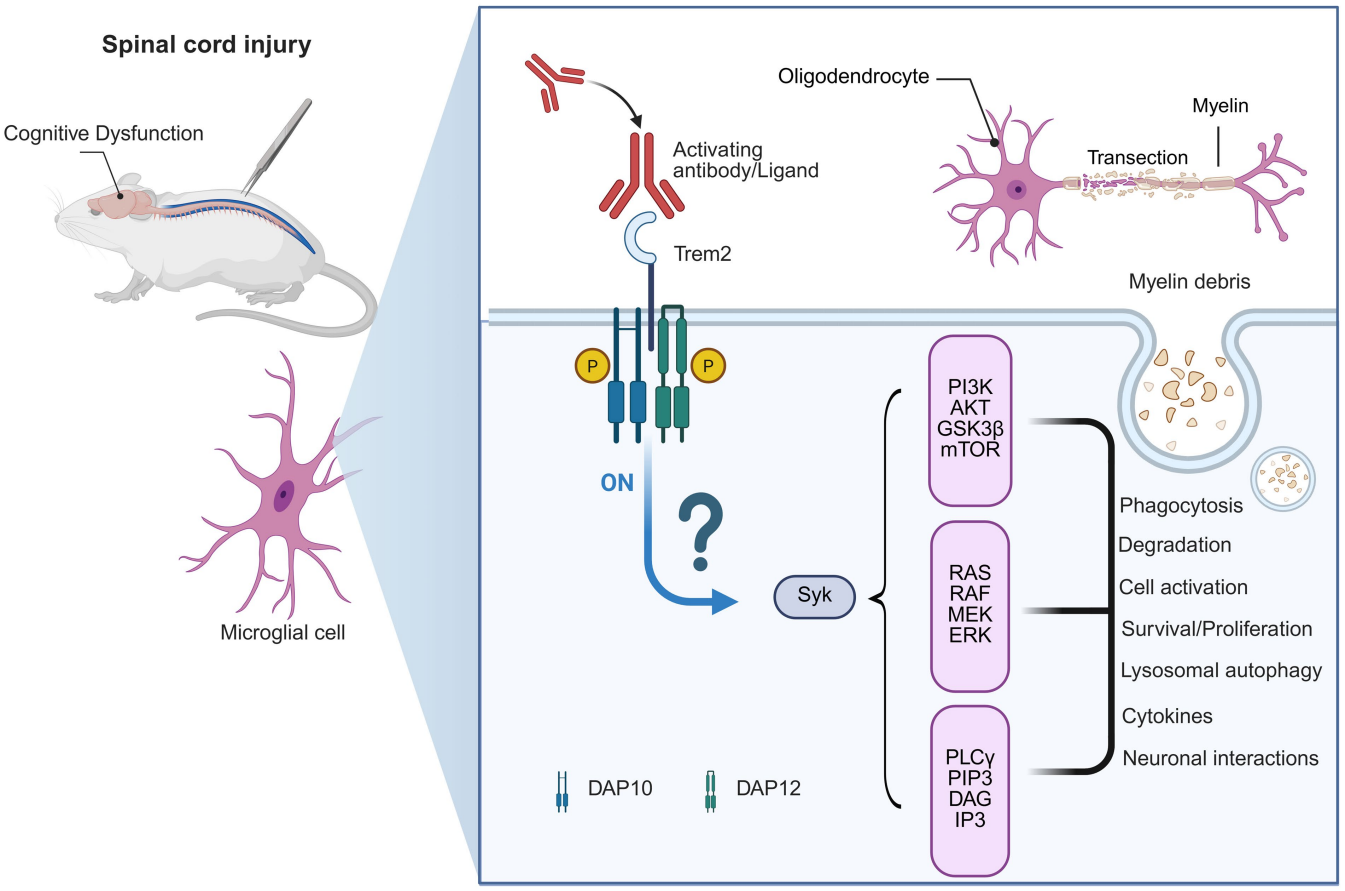
Hypothetical mechanism of TREM2 in spinal cord injury-induced cognitive dysfunction. This figure illustrates a hypothetical model. Although microglial activation after spinal cord injury (SCI) is well established, the specific pathways through which these cells exert their functions remain unclear. This diagram shows a potential mechanism for cognitive dysfunction following SCI. While research on TREM2’s role in SCI is limited, we hypothesize that TREM2 may play a role in SCI-related cognitive impairment. SCI leads to myelin debris produced and microglial activation. TREM2 could be involved in clearing myelin debris at the injury site, and may also contribute to other changes in the brain following SCI, potentially influencing neuroinflammation, neuronal survival, and cognitive function, similar to its role in Alzheimer’s disease.

Microglial TREM2 was identified as a critical modulator for neuroinflammation and cognitive function in AD, whereas its involvement in SCI remains largely unknown (Gao et al., 2023; Zhao T. et al., 2025). SCI can induce microglial activation not only at the lesion site but also in supraspinal regions, which may have a dual effect in chronic inflammation and neuronal damage (Milich et al., 2021; Gong et al., 2023; Skinnider et al., 2024). Given TREM2’s role to modulate microglial phagocytic activity, inflammatory cytokine release, and synaptic remodeling (Kobayashi et al., 2016; Li et al., 2022; Li Z. et al., 2024; Yan et al., 2022), it is reasonable to speculate that the cognitive performance following SCI may also be modulated by TREM2 (Figure 2).

Data from AD support the idea that TREM2 has been shown to drive the conversion of microglia into DAM that are involved in the clearance of cellular debris and maintenance of the synaptic integrity, which are critical for improving cognitive capacity (Nugent et al., 2020; Wu et al., 2022; Parhizkar et al., 2023; John et al., 2025; Zhu et al., 2025; Wu et al., 2022; Parhizkar et al., 2023; John et al., 2025; Zhu et al., 2025). It should be stressed that these are hypothesis-driven extrapolations, as there is little experimental or clinical SCI-specific cognition-related data on TREM2. Therefore, the discussion below should be read as a conceptual comparison rather than evidence-based. If we extend these findings to SCI, it can be speculated that TREM2-related microglial responses may reduce maladaptive inflammation, ameliorate synaptic plasticity in cortical and hippocampal circuits, and ultimately prevent attention and memory deficits. Such a mechanistic model offers a new insight into the cellular substrates of SCI-induced cognitive deficiency.

## 4 Therapeutic implications of targeting TREM2 in SCI

Investigating TREM2 in the context of SCI also opens avenues for therapeutic innovation (Gao et al., 2023; Zhao T. et al., 2025). By targeting TREM2, it may be possible to modulate microglial activity in a way that both reduces chronic neuroinflammation and enhances cognitive resilience (Yan et al., 2022; Li Z. et al., 2024). These approaches can potentially complement current rehabilitation paradigms, providing precision medicine guidelines. In addition, when AI is used for analyzing multimodal datasets (single cell transcriptomics, neuroimaging, and clinical cognitive outcomes), it will help speed up the discovery of these TREM2-related mechanisms/facilitate the potential intervention points that could open vistas towards next-generation therapies (Kalaga and Ray, 2025; Liu X. et al., 2025). While most research stresses protective roles of TREM2, other studies also indicate that TREM2 activation may potentially exacerbate inflammation or impair recovery after SCI (Zhao T. et al., 2025). These findings highlight the need for context-specific investigations.

## 5 AI-assisted strategies for discovery and translation

### 5.1 Computational approaches for mechanism discovery and therapy design

With these mechanistic understandings in hand, we now consider AI as a potential catalyst for discovery and translation (Liu et al., 2024b, 2025c). AI offers great potential to expedite the discovery of microglial targets in cognitive impairment from SCI (Kalaga and Ray, 2025). Leveraging on big-data resources derived from single-cell transcriptomics, proteomics, brain imaging, and cognitive clinical data can connect TREM2-high expressed microglial subpopulations with inferred functional states and predictions of their potential consequence for synapse plasticity and neural circuit performance (Liu X. et al., 2025). Such analyses may reveal novel cellular and molecular pathways connecting SCI-induced neuroinflammation with cognitive impairment (Table 1).

**Table 1:** TREM2’s role in Alzheimer’s disease (AD)/spinal cord injury (SCI) and AI-assisted opportunities.

Aspect	Alzheimer’s disease (AD)	Spinal cord injury (SCI)	AI-assisted opportunities	Key references
Pathological context	Aβ/Tau deposition; synaptic loss	Inflammatory cascade, brain–spinal crosstalk, circuit remodeling	AI can integrate imaging + omics to identify shared pathological features	Mathys et al. (2023), Peña-Bautista et al. (2024), Leuzy et al. (2024), and Craig et al. (2017)
Microglial response	TREM2 drives DAM phenotype, enhances clearance, neuroprotection	Widespread activation; TREM2 may regulate inflammation and plasticity	AI can identify TREM2 + subpopulations and track state transitions	Jiang et al. (2014), Li et al. (2022), Shi Q. et al. (2022), Fracassi et al. (2023), and John et al. (2025)
Downstream pathways	PI3K/Akt, Wnt/β-catenin, SYK, mTOR	Potential involvement in inflammation, lipid metabolism, autophagy	AI-based network modeling can predict novel therapeutic targets	Nugent et al. (2020), Li et al. (2022), Peng et al. (2023), Zhao T. et al. (2025), and John et al. (2025)
Cognitive outcomes	Memory and executive decline	Deficits in attention, memory, processing speed	AI predictive models can combine imaging + clinical data for prognosis	Bäckman et al. (2004), Jure et al. (2022), Li Z. et al. (2024), and Welkamp et al. (2024)
Therapeutic prospects	TREM2-targeted drugs under development	Early-stage exploration, limited validation	AI-driven drug repurposing, precision rehab (VR, BCI)	Jia et al. (2025), Liu X. et al. (2025); Noh et al. (2025), and John et al. (2025)

In addition to mechanism discovery, AI has potential in guiding the direction for therapeutic development (Liu et al., 2020; Fang et al., 2022). Deep learning and computational modeling can promote high-throughput virtual screening of small molecules or biologicals targeting TREM2 activity for prioritizing candidates with desirable efficacy and safety profiles (Fang et al., 2022; Liu X. et al., 2025). Furthermore, AI-based predictive models can predict patient-specific cognitive trajectories to design precision interventions for each SCI patient according to molecular and clinical biomarkers (Möhle et al., 2021; Trivedi et al., 2023; Calderone et al., 2024; Kale et al., 2024; Bitsch et al., 2025; Noh et al., 2025).

AI can also strengthen cognitive rehabilitation approaches (Lee et al., 2023; Liu et al., 2025a). Brain–computer interfaces, virtual reality platforms, and adaptive neurofeedback systems can be integrated with AI models to dynamically adjust training programs according to real-time cognitive performance and microglial biomarker profiles, leading to adaptive neurofeedback systems modifying training programs (Lee et al., 2023; Kishikawa et al., 2024; Yoo et al., 2024; Liu et al., 2025b; Noh et al., 2025). These approaches could enable tailored interventions to the enhancement or suppression of neural plasticity and immune-mediated responses that may be dovetailed in an attempt to best optimize individual recovery therapies (Kale et al., 2024). For example, a group proposed an AI-based motion analysis for rehabilitation in patients with SCI (Lee et al., 2023). Another group verified the machine learning models for prediction after cervical SCI (Kishikawa et al., 2024), whereas some researchers used neural networks to detect neuropathic pain signatures following SCI (Deulofeu et al., 2024). In TREM2-related biology, some studies applied interpretable deep learning to represent microglial activation states in AD and is a methodological blueprint for SCI (Trivedi et al., 2023). In contrast to classical statistics, these methods enable to combine high-dimensional data and to make prediction for individual-patient, that constitutes an exceptional feature.

Collectively, AI-augmented methods provide a complementary environment to connect basic mechanistic insights with translational and clinical practices (Deng et al., 2020; Perosa et al., 2021; Pancholi et al., 2024; Tao et al., 2024). By combining both computational resources and neuroimmunological experience, these approaches could hasten the discovery of new TREM2-directed interventions and enhance cognitive recovery in SCI patients.

### 5.2 Challenges and future directions in AI-driven neuroimmunology

Although research on TREM2 has revealed how microglia are regulated, some obvious challenges still exist when applying these findings to understand cognitive impairment after SCI (Gao et al., 2023; Zhao T. et al., 2025). First, direct experimental evidence of a role for TREM2 in cognition following SCI is lacking (Zhao T. et al., 2025), and knowledge remains scarce and needs to be filled by targeted preclinical studies. Second, the heterogeneity of SCI patients (e.g., level and severity of lesion, age, and comorbidities) leads to a lack of common biomarkers and therapeutic targets (Welkamp et al., 2024). Third, current bio-verification of computerized AI-based predictions is only available in the context of standardized (multi-)modal data types and more complicated computational processing pipelines that have not been broadly validated across the SCI research community (Deulofeu et al., 2024; Habibi et al., 2024; Daungsupawong and Wiwanitkit, 2025). Fourth, AI also faces barriers, including limited availability of high-quality multimodal datasets, lack of reproducibility across cohorts, and difficulties in regulatory validation for clinical use (Liu et al., 2024a).

More work remains for modest investigation of the TREM2 role with post-SCI cognitive outcomes, which may be carried out in animal models, single cells at the molecular level, as well as patients by means of longitudinal clinical studies (Španić Popovački et al., 2023; Zhang et al., 2023, 2025). Furthermore, interdisciplinary approaches fusing neuroimmunology with cognitive neuroscience and computational modelling are required to help design predictive personalized treatments (Pereira et al., 2022; Kale et al., 2024). For instance, AI-driven drug discovery and patient stratification, as well as adaptive rehabilitation programs, have emerged as very promising options to translate mechanistic insights into clinical interventions (Fang et al., 2022; Liu X. et al., 2025). The field can thus progress toward more specific approaches to treat cognitive dysfunction in SCI patients.

## 6 Summary and outlook

Cognitive impairment is an important but relatively unappreciated complication of SCI and has far-reaching effects on patient outcome and quality of life. Given that microglial TREM2 is a known modulator of neuroinflammation and cognition in AD, it provides an attractive target for mechanistic studies relevant to SCI. Merging insights from AD and SCI research conceptualizes how TREM2-dependent microglial responses can affect attention, memory, and executive function following CNS injury.

AI could further improve this framework by helping to define TREM2-related pathways, drug discovery, and personalized cognitive rehabilitation. Connecting neuroimmunology, the cognitive sciences, and AI-led therapeutics, we provide a perspective on new avenues for both mechanistic knowledge and translational impact. Prospective investigations in this category will be able to improve cognitive status and the general recovery process for SCI by focusing on TREM2.
